# Angiography-Assisted Cone-Beam CT-Guided Radiofrequency Ablation for Hepatocellular Carcinoma: Single-Center Workflow and Early Outcomes

**DOI:** 10.3390/diagnostics15222898

**Published:** 2025-11-15

**Authors:** Jung Ui Hong, Soon Gu Cho, Kyu Hong Lee, Ji Hoon Noh, Ro Woon Lee

**Affiliations:** Department of Radiology, Inha University College of Medicine, 27 Inhang-ro, Jung-gu, Incheon 22332, Republic of Korea; ghdwjddml3@naver.com (J.U.H.);

**Keywords:** radiofrequency ablation, hepatocellular carcinoma, Image-guided ablation, Cone-beam CT with intra-arterial angiography

## Abstract

**Background:** Conventional CT- or US-guided radiofrequency ablation (RFA) for hepatocellular carcinoma (HCC) often limits repeat contrast-enhanced imaging and provides suboptimal intraprocedural conspicuity, which can hinder precise targeting and margin assessment. **Purpose:** To describe a single-center angiography-assisted cone–beam CT (angio-CBCT) RFA workflow and report early outcomes versus an institutional conventional CT-guided cohort. **Materials and Methods:** In this IRB-approved single-center retrospective study, consecutive patients underwent angio-CBCT-guided RFA for HCC (n = 14). Selective intra-arterial injections (≈20–40 mL iodinated contrast per CBCT) through a 5-Fr catheter permitted multiple intraprocedural CBCT acquisitions for targeting, verification, and endpoint assessment under general anesthesia. Primary outcomes were technical success, early local recurrence, and complications (CTCAE v6.0). For a secondary imaging analysis, within-patient change in lesion conspicuity (ΔHU = HU_tumor − HU_liver) from preprocedural contrast-enhanced CT to intraprocedural imaging was compared in available cases (angio-CBCT n = 12; conventional CT n = 13). Descriptive statistics were used. **Results:** Angio-CBCT RFA achieved technical success in 14/14 (100%) procedures; early local recurrence was 0/14 (0.0%); and one complication occurred (1/14, 7.1%; Grade 3). Intraprocedural refinements included immediate re-ablation in 3/14 (21.4%) and electrode repositioning in 2/14 (14.3%), with on-table detection of an additional lesion in 1/14 (7.1%). In the institutional conventional cohort, technical success was 19/20 (95.0%), early local recurrence 2/20 (10.0%), and complications 0/20 (0%). Lesion conspicuity improved with angio-CBCT (median ΔHU 290.1 HU, n = 12) but not with conventional CT (−10.5 HU, n = 13). **Conclusions:** Angio-CBCT-guided RFA for HCC is feasible and safe and enables repeatable, low-volume contrast-enhanced intraprocedural imaging that supports precise targeting, verification, and timely refinements. Early outcomes and markedly improved lesion conspicuity suggest potential advantages over conventional CT-guided workflows and warrant prospective validation in larger cohorts.

## 1. Introduction

Hepatocellular carcinoma (HCC) is one of the most common malignancies worldwide and remains a leading cause of cancer-related mortality; its incidence and mortality are increasing in both Western and Asian countries [1]. In patients with early-stage HCC who are not suitable candidates for surgery or transplantation, local ablative therapies provide a curative-intent alternative. Radiofrequency ablation (RFA) has become a widely accepted standard therapy for tumors ≤3 cm, offering survival outcomes comparable to surgical resection while avoiding the morbidity of surgery. Several studies have shown that RFA yields high complete response rates and low complication rates, and in carefully selected patients overall survival is similar to that of surgical resection [2,3]. 

The success of percutaneous RFA is critically dependent on accurate electrode placement and achieving an adequate ablative margin. Early imaging studies have demonstrated that an ablative margin <5 mm is a significant predictor of local tumor progression and overall recurrence, whereas achieving a ≥5 mm margin reduces the risk of recurrence [4]. Therefore, intraprocedural assessment of the ablation zone and margin is essential to ensure complete tumor necrosis.

Conventional guidance for RFA relies on ultrasonography (US) or computed tomography (CT). US is portable and radiation-free but often fails to visualize tumors in challenging locations and is operator dependent; moreover, intraprocedural hyperechoic gas obscures the ablation zone and hampers margin assessment [5]. CT offers excellent cross-sectional detail, yet isoattenuating HCCs are difficult to delineate without contrast, and multiphase imaging typically requires ~100–120 mL per run—≥200 mL for pre- and post-ablation scans—limiting repeat acquisitions within a single procedure [6].

Although CT hepatic arteriography uses <20 mL of contrast per scan and allows multiple acquisitions, it requires catheter placement in the hepatic artery and transfer between the angiography suite and CT room, which is logistically challenging [7]. To improve tumor conspicuity and overcome US limitations, fusion imaging techniques that overlay CT or MR data onto real-time US have been developed; these methods enhance lesion detectability for poorly conspicuous tumors and reduce false-positive detections. Nevertheless, fusion imaging still depends on US for needle placement and remains prone to gas artifacts during RFA [5,8]. 

Angiography-assisted cone-beam CT (CBCT) has emerged as a promising solution to these limitations. In the context of transarterial chemoembolization (TACE), CBCT provides three-dimensional volumetric imaging that increases the sensitivity for detecting hypervascular tumors [9]. This technology allows real-time overlay of reconstructed 3-D images on fluoroscopy and uses small doses of contrast. Selective hepatic arteriography with CBCT requires only 15–30 mL of iodinated contrast per acquisition, markedly enhancing tumor-to-liver contrast and permitting multiple scans without exceeding the maximum allowable contrast dose [6]. To address the logistical challenge of transferring patients between the angiography suite and the CT room, techniques employing cone-beam CT within the angiography suite—rather than multi-detector CT in a separate CT room—have been introduced [10], and recent reports have described CBCT as a guidance modality for RFA [11,12].

Building on these advances, we developed a new angiography-assisted CBCT-guided RFA technique for HCC. This method involves selective catheterization of the celiac or hepatic artery with a 5-Fr catheter and infusion of 20–40 mL of contrast during CBCT acquisition, producing high tumor-to-liver contrast and enabling repeated imaging for targeting, electrode repositioning and immediate assessment of the ablation zone. By integrating CBCT guidance within the angiography suite and using general anesthesia with apnea, the technique overcomes the visualization challenges of US and the contrast limitations of conventional CT. Here we describe our initial 2-year experience treating 14 patients with HCC using angiography-assisted CBCT-guided RFA, focusing on technical feasibility, safety and preliminary efficacy.

## 2. Materials and Methods

### 2.1. Study Design and Patient Selection

This study included all consecutive patients who underwent angiography-assisted cone-beam CT (CBCT)-guided radiofrequency ablation (RFA) for hepatocellular carcinoma between 1 October 2023 and 31 August 2025 at our institution. In addition, a benchmark group treated between 1 July 2022, and 31 August 2025, was also included for comparison. Treatment modality selection and candidacy for RFA were determined in a multidisciplinary setting after review of pre-procedural imaging, including ultrasound (US), liver multiphase CT, liver MRI, and contrast-enhanced ultrasound (CEUS).

The benchmark group included patients treated between 1 July 2022, and 31 August 2025, who received conventional RFA techniques, specifically US-guided, CT-guided, or intraoperative RFA. The procedures for this group were performed according to standard institutional protocols, which involved the use of either conventional CT or ultrasound for guidance. The study protocol was approved by the Institutional Review Board of Inha University Hospital (IRB No. 2025-10-002, Date of Approval: 1 October 2025). Given the retrospective design, exclusive use of de-identified clinical and imaging data generated during standard care, and the absence of any additional interventions or risks to patients, the IRB determined that the study involves no more than minimal risk and that obtaining consent from all eligible subjects would be impracticable. Therefore, the requirement for written informed consent was waived.

### 2.2. Periprocedural Preparation and Anesthesia

Patients were admitted 1–2 days before the planned RFA. All procedures were performed in the angiography suite under general anesthesia to minimize respiratory motion and pain that could interfere with targeting and ablation. Patients were positioned supine on the C-arm table, and the endotracheal tube and intravenous lines were deliberately routed to accommodate unobstructed CBCT gantry rotation. General anesthesia was induced and maintained in collaboration with the anesthesiology team, with the airway secured and physiologic parameters continuously monitored in accordance with institutional protocols.

### 2.3. Hepatic Angiography, and Angio-CBCT Acquisition

Under ultrasound guidance, the tumor location and the common femoral artery (CFA) were identified. After sterile preparation and draping, the CFA was punctured and an introducer angiosheath was inserted. Using a 0.035-inch guidewire and a 5-Fr RH-shaped angiographic catheter, the catheter was selectively positioned in an artery suitable for angiography—the celiac axis, common hepatic artery, proper hepatic artery, or the right or left hepatic artery; when selective access with a 5-Fr catheter was suboptimal, a microcatheter was additionally used. With intra-arterial contrast injection, digital subtraction angiography (DSA) was performed to assess vascular anatomy and hemodynamics and to determine the degree and timing of HCC enhancement. On the basis of the angiographic findings, the contrast dose and scan timing were selected, and the first angiography-assisted CBCT (angio-CBCT) was acquired. The C-arm rotation time was 6 s. To synchronize CBCT acquisition with peak arterial enhancement of hepatocellular carcinoma (HCC), a case-specific scan delay was applied after the start of contrast injection. During the 10–15 s CBCT acquisition, mechanical ventilation was briefly suspended under anesthesiology supervision to induce apnea and minimize motion artifacts. The contrast volume per CBCT ranged from 20 to 40 mL.

### 2.4. Electrode Placement and Multi-Modal Needle Guidance

Following the first angio-CBCT, percutaneous advancement of the RFA electrode toward the target HCC was performed. A commercially available RFA generator (VIVA Multi^®^; STARmed, Goyang, Republic of Korea) with internally cooled separable clustered electrode (Octopus^®^ electrodes: STARmed, Goyang, Republic of Korea) was used. Needle guidance leveraged all available modalities, including gray-scale ultrasonography, CEUS, the angio-CBCT dataset, and the vendor’s needle guidance software (Artis Q Ceiling, Siemens healthineers, Forchheim, Germany) in combination with a C-arm–mounted laser guidance kit. The CBCT volume was used to (i) confirm the exact 3-D location of the target lesion, (ii) screen for additional occult lesions, and (iii) delineate non-target critical structures to be avoided (intrahepatic vessels and bile ducts, and adjacent organs such as the gallbladder, diaphragm, colon, kidney, and heart). When the tumor abutted vulnerable structures and thermal injury was a concern, artificial ascites infusion was applied under US guidance to create a protective peritoneal fluid buffer before electrode advancement and ablation [13].

For the benchmark group, conventional methods were used. CT-guided RFA relied on standard CT imaging for electrode placement, while US-guided RFA utilized real-time ultrasound to direct the electrode. In intraoperative RFA, needle guidance was performed based on real-time ultrasound and direct visualization of mass

### 2.5. Verification CBCT and Electrode Repositioning

After initial electrode placement, a second angio-CBCT was acquired using the same contrast-timing protocol (including brief apnea during the 10–15 s acquisition) to evaluate (i) whether the electrode tip and active segment were optimally seated within the target tumor, (ii) whether the anticipated ablation zone would fully encompass the lesion with an adequate margin, and (iii) whether there was any risk of thermal injury to adjacent non-target structures. If positioning was deemed suboptimal, the electrode was repositioned and a third angio-CBCT was obtained; this check-adjust-confirm loop was repeated until satisfactory targeting and safety margins were achieved. In most procedures, the intended electrode trajectory was achieved with a single puncture. In some cases, one or more repositioning attempts were required, with position verified by repeat CBCT.

### 2.6. Ablation Delivery and Post-Ablation Imaging

Thermal ablation was performed and continued until the tissue surrounding the electrode was fully encompassed by the ablation zone. Immediately after each ablation, a post angio-CBCT was acquired using the same contrast-timing and apnea protocol to evaluate treatment effect. Successful ablation was defined as meeting both criteria: (1) the ablation zone completely covered the entire pre-treatment tumor region, and (2) any tumor enhancement visible on the pre-procedural angio-CBCT was absent on the post-procedural angio-CBCT. When the ablation zone was insufficient or residual enhancement persisted, the electrode was repositioned and additional ablation was performed.

This sequence was repeated as needed until ablation was successful. In most procedures, the initial ablation sufficed; some required additional ablations. Software-based confirmation tools were not available during the study period and were not used; needle position and margins were assessed visually on pre- and immediate post-ablation CECT/angio-CBCT. This visual assessment allowed for differentiation between residual tumor and hyperemia by comparing the enhancement patterns and morphology between the two areas.

### 2.7. Immediate Safety Assessment and Procedure Completion

Immediate complications were assessed during the procedure. If post–angio-CBCT demonstrated contrast extravasation suggestive of hemorrhage, immediate TAE via the indwelling catheter was planned. After successful ablation was confirmed, to prevent needle-tract seeding the generator’s tract-ablation mode was activated and the electrode was withdrawn while maintaining approximately 70 °C along the tract. Hemostasis of the CFA access site was achieved with a vascular closure device, the angiographic catheter and sheath were removed, and general anesthesia was discontinued by the anesthesiology team.

### 2.8. Endpoints

The primary endpoint was the technical success (and residual disease) rate of angiography-assisted CBCT-guided RFA. Secondary endpoints included (i) time to local recurrence (TLR), (ii) occurrence of procedure-related complications, (iii) the number of electrode repositioning events after the first placement, and (iv) the number of additional ablations required after the first ablation.

Endpoints for image-guided tumor ablation were defined according to previously published consensus recommendations [14].

In the angio-CBCT cohort, technical success was confirmed by intraprocedural cone-beam CT. For the benchmark cohort, the margin determination varied by imaging modality: in the US-guided cohort, it was determined by the high-echoic bubbles from RFA; in the CT-guided cohort, by the peripheral hyperemia enhancement seen on contrast-enhanced CT. In the intraoperative cohort, as ultrasound was always used. Based on these findings, technical success was defined as meeting both criteria (1) and (2), as in the angio-CBCT RFA cohort.

Local recurrence was assessed on follow-up CT or MRI by review of radiology reports; any imaging evidence of residual or recurrent tumor at the ablation zone was classified as local recurrence. TLR was defined as the time from the index ablation to imaging-confirmed local recurrence. Patients without recurrence were censored at the last imaging date or 30 October 2025 (administrative censoring), whichever occurred first.

### 2.9. Complication

Electronic medical records were screened for procedure-related complications through 90 days after treatment, and all adverse events were graded according to CTCAE version 6.0 [15].

### 2.10. Quantitative Analysis of Enhancement and Contrast Agent Use

From the pre-ablation angio-CBCT datasets, the degree of HCC enhancement was quantified by measuring Hounsfield units (HU) within the arterial-phase enhancing tumor and within adjacent non-enhancing normal liver parenchyma, and the values were compared (e.g., tumor HU, liver HU, and ΔHU = HU_tumor − HU_liver).

For each procedure, we recorded the iodinated contrast material volume (mL), the total number of CT acquisitions, and the number of contrast-enhanced CT acquisitions, and compared these metrics between the angio CBCT group and the conventional CT group. In addition, all primary and secondary outcomes were benchmarked against a conventional RFA cohort from the same institution that did not use the angio-CBCT technique—specifically US-guided RFA, CT-guided RFA, and intraoperative RFA—performed between 1 July 2022, and 31 August 2025. Identical endpoint definitions and abstraction methods were applied across cohorts. Additionally, cases deemed inappropriate outliers for this analysis were excluded: RFA after TACE (lipiodol-guided lesions), ultrasound-only RFA, intraoperative RFA, and cases lacking a pre-ablation contrast-enhanced CT.

### 2.11. Statistical Analysis

Given the small sample size, no hypothesis testing or multivariable modeling was performed. Results are presented as descriptive statistics: continuous variables as mean ± SD or median (IQR) and categorical variables as counts (percentages). For key binary outcomes (technical success, complications, local recurrence), exact (Clopper–Pearson) 95% confidence intervals were calculated. Time-to-event endpoints (TLR) were summarized using the Kaplan–Meier method with numbers at risk

## 3. Result

### 3.1. Patient Demographics

Baseline characteristics were broadly comparable between the angio CBCT RFA cohort (n = 14) and the benchmark cohort (n = 20). Median age was 71 years (range, 52–85) versus 70 (52–82), and 57.1% versus 85.0% were male. Most patients were Child–Pugh class A (85.7% vs. 100%); no class C occurred in either cohort. All tumors were HCC. Tumor burden was low and similar: median largest tumor 1.6 cm (0.9–2.5) versus 1.9 cm (0.5–3.2); most patients had a single lesion (85.7% vs. 90.0%); tumors ≥3 cm were rare (0% vs. 5%). Baseline characteristics are summarized in Table 1.

### 3.2. Procedural Outcomes

Procedural outcomes in the angio CBCT-guided cohort are summarized in Table 2. Technical success was achieved in 14/14 (100.0%) sessions, and no local recurrence (0/14, 0.0%) was recorded within this dataset. Complications occurred in 1/14 (7.1%). Intra-procedural refinements were frequent: target repositioning in 2/14 (14.3%), immediate re-ablation in 3/14 (21.4%), and detection of an additional new mass in 1/14 (7.1%). These descriptive results are listed in Table 2.

### 3.3. Comparative Outcomes (CBCT vs. Conventional RFA)

Compared with conventional RFA, the angio CBCT RFA group showed numerically higher technical success (100.0% vs. 95.0%), lower local recurrence (0.0% vs. 10.0%), and a small increase in complications (7.1% vs. 0.0%); however, these differences were not statistically significant in this sample (Technical success (Fisher’s exact *p* = 1.000). Local recurrence (*p* = 0.501). Complications (*p* = 0.412). Median [IQR] imaging follow-up was 460 days [205–636] for angio-CBCT and 967 days [801–1076] for conventional. Using a time-to-event approach (Kaplan–Meier), local control at 12 months was 100% in both groups. At 24 months, local control was 100% (95% CI NE, no events; at-risk n = 1) for angio-CBCT and 94.1% (95% CI 65.0–99.1) for conventional. (Deaths were not modeled as competing events). Comparative outcomes are listed in Table 3.

### 3.4. Secondary Imaging Outcome—ΔHU

**Figure 1:**
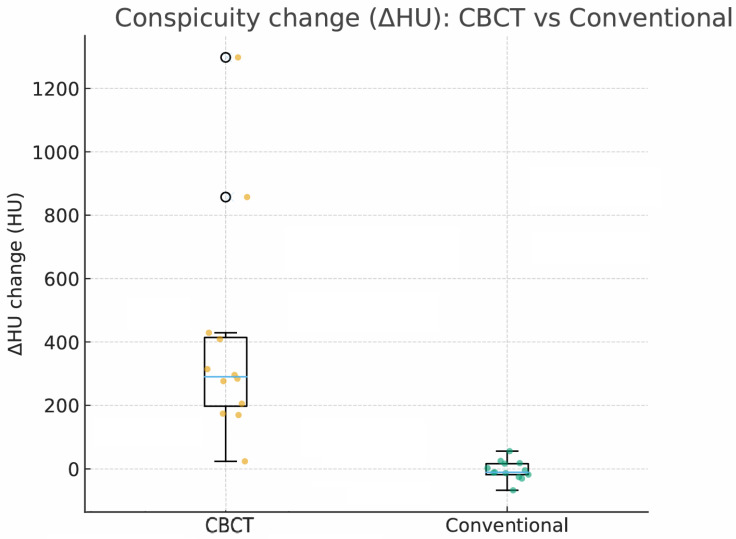
Change in lesion conspicuity (ΔHU) from preprocedural CT to intraprocedural imaging. Boxes show the interquartile range with the median line; dots represent individual patients (orange dot = CBCT group patients, green dot = conventional group patients). CBCT demonstrated higher ΔHU on distributional summaries than conventional CT in this exploratory analysis [CBCT: 290.1 HU (IQR, 197.5–414.1; n = 12); conventional: −10.5 HU (IQR, −17.9–16.5; n = 13)].

Using within-patient change from baseline, angio CBCT-guided imaging showed higher lesion conspicuity than conventional CT guidance. The ΔHU change (intraprocedural − preprocedural) was 290.1 HU (IQR, 197.5–414.1) in the CBCT group (n = 12) versus −10.5 HU (IQR, −17.9–16.5) in the conventional group (n = 13) (Figure 1). Given the exploratory nature and limited sample size, formal inference was minimized and no multiplicity adjustment was applied. Of the overall cohort (CBCT n = 14; conventional n = 20), the ΔHU analysis excluded prespecified outliers—RFA after TACE (lipiodol-guided lesions), ultrasound-only RFA, intraoperative RFA, and cases without pre-ablation contrast-enhanced CT that precluded lesion-HU measurement—yielding the analysis subset of n = 12 and n = 13, respectively.

**Figure 2:**
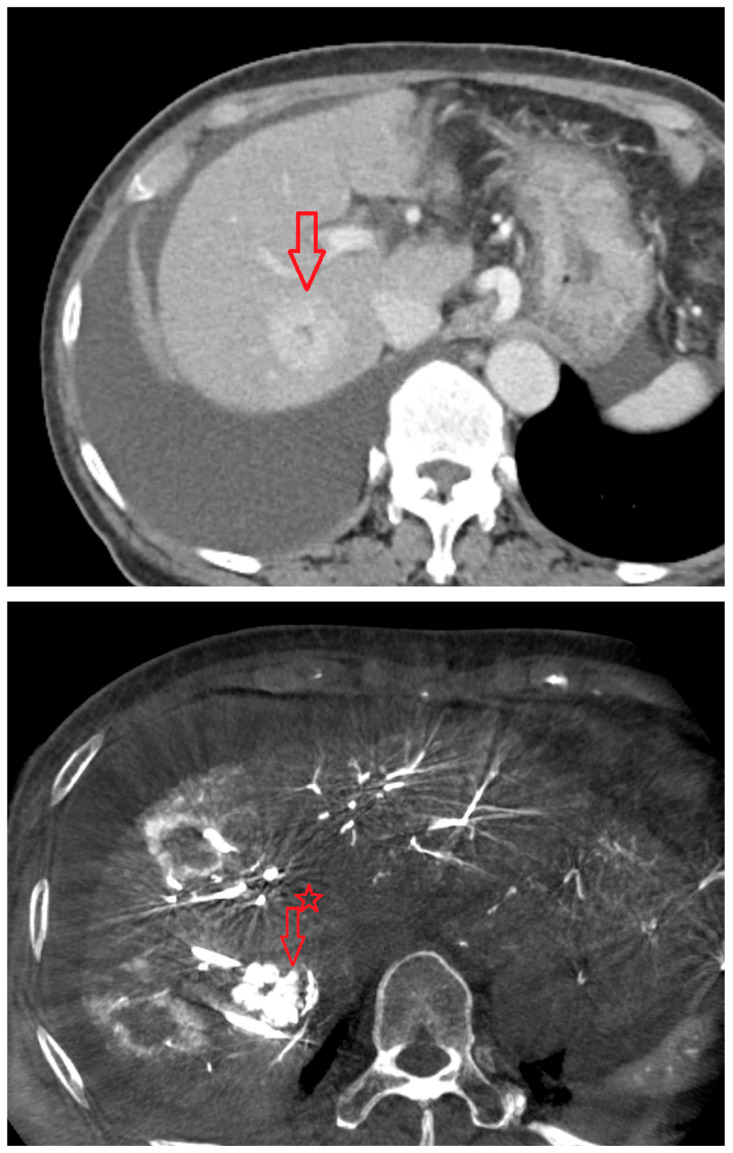
CT images from a patient with hepatocellular carcinoma undergoing angio CBCT-guided RFA. (**Top**): pre-procedural CT (portal venous phase; lesion 128.7 HU, red arrow = HCC mass). (**Bottom**): intraprocedural angio CBCT (lesion 1499.7 HU, red arrow with star = HCC mass). ΔHU = 1298.0 HU. (intra lesion − intra background) − (pre lesion − pre background).

**Figure 3:**
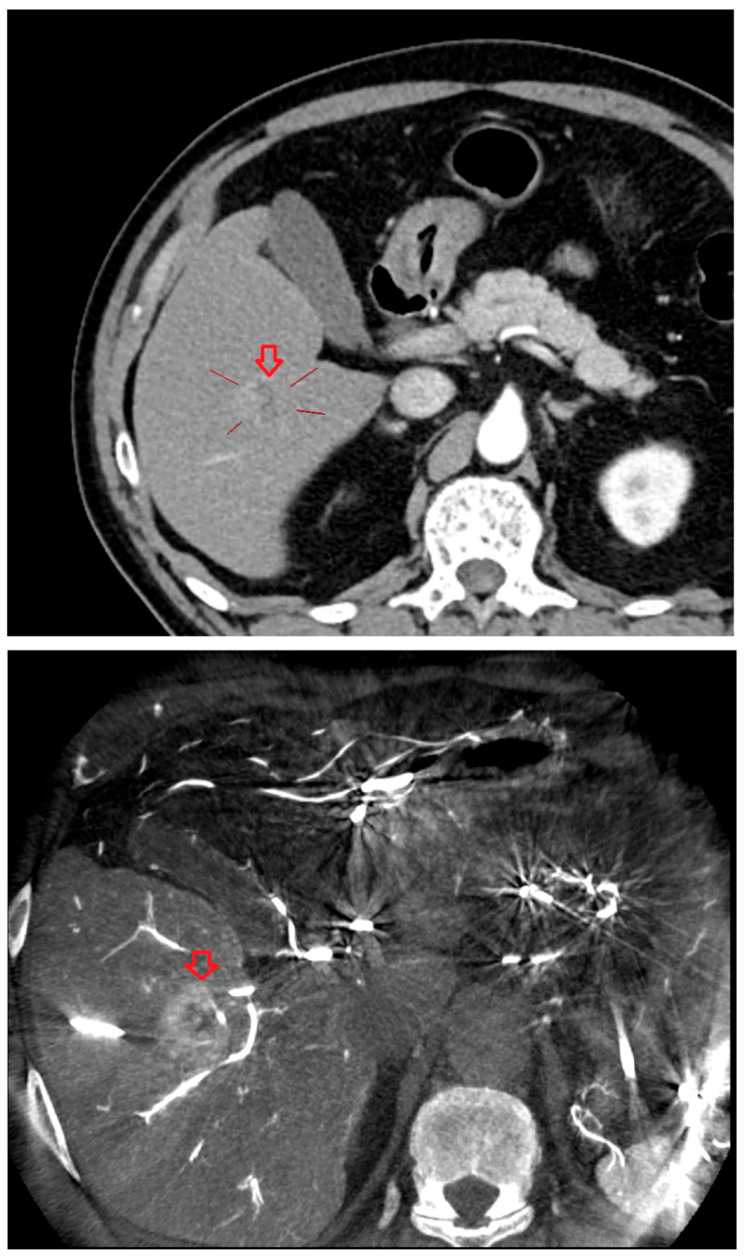
CT images from other patient with hepatocellular carcinoma undergoing angio CBCT-guided RFA. (**Top**): pre-procedural CT (arterial phase). Red arrow and line = HCC mass (lesion 74.29 HU). (**Bottom**): intraprocedural angio CBCT. Red arrow = HCC mass (lesion 368.49 HU). ΔHU = 173.9 HU. (intra lesion − intra background) − (pre lesion − pre background).

**Figure 4:**
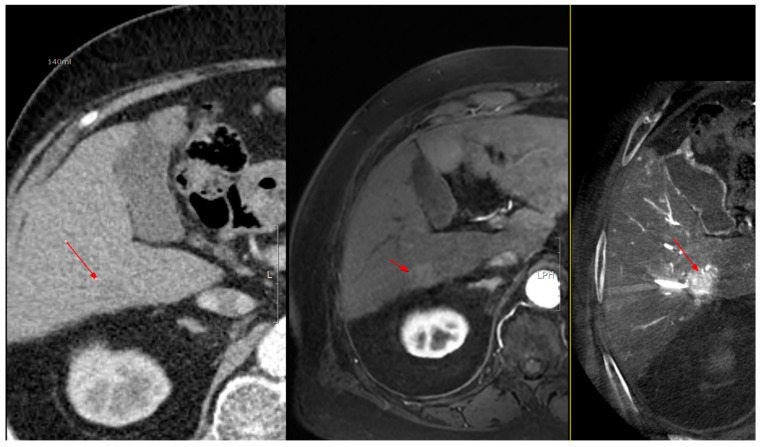
Images from another patient with hepatocellular carcinoma undergoing angio CBCT-guided RFA. All red arrows indicate the HCC mass. (**Left**): pre-procedural CT (portal phase). (**Middle**): pre-procedural MRI (arterial phase). (**Right**): intraprocedural angio CBCT. (Intra lesion − Intra background = 432.7 HU).

**Figure 5:**
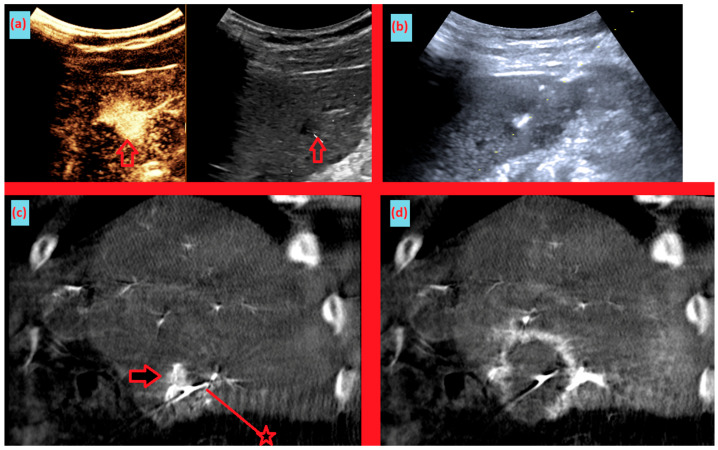
Images from another patient with hepatocellular carcinoma undergoing angio CBCT-guided RFA. (**a**) Pre-ablation CEUS and B-mode ultrasound demonstrating discordance between the CEUS-enhancing area and the hypoechoic lesion on B-mode. (arrow = mass) (**b**) During RFA, ultrasound-guided needle insertion was performed, targeting the hypoechoic lesion on B-mode. (**c**) Sagittal reformatted angio CBCT obtained after the initial needle placement shows the needle positioned inferior to the enhancing tumor. After review, the needle was repositioned slightly superiorly. (arrow = mass, line with star = RFA needle) (**d**) Sagittal reformatted angio CBCT after ablation demonstrates an adequate ablation zone with complete loss of lesion enhancement, consistent with complete ablation.

**Figure 6:**
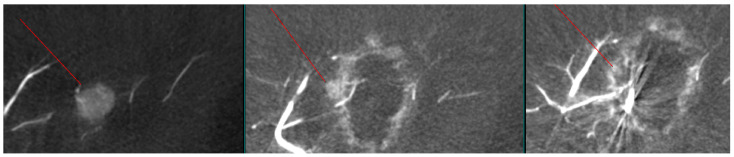
Images from another patient with hepatocellular carcinoma undergoing angio-CBCT-guided RFA. (**Left**): Pre-ablation angio-CBCT shows an enhancing mass in the left lobe (line marking). (**Middle**): Post-ablation angio-CBCT shows an incomplete ablation margin with hyperemia, and a small enhancing nodule attached to the margin, likely representing a residual mass (line marking). (**Right**): Post re-ablation angio-CBCT shows a complete ablation margin with the disappearance of the residual mass (line marking).

As shown in Figure 1, angio-CBCT allows for higher visibility of the target mass, as reflected in the ΔHU values. This can be visually confirmed in several cases. Representative cases in Figure 2 and Figure 3 demonstrate how the target mass is much more conspicuous to the operator on angio-CBCT compared to pre-procedural CT. In Figure 4, a mass, which was nearly invisible on pre-procedural CT, was identified through angio-CBCT, aiding the targeting process. Figure 5 shows a case where a mass with discordance between USG and CEUS was confirmed using angio-CBCT, leading to needle repositioning and achieving complete ablation. Finally, Figure 6 illustrates a case where a residual tumor was identified on angio-CBCT and re-ablation resulted in complete ablation.

### 3.5. Contrast Use and Number of Contrast-Enhanced Acquisitions

**Figure 7:**
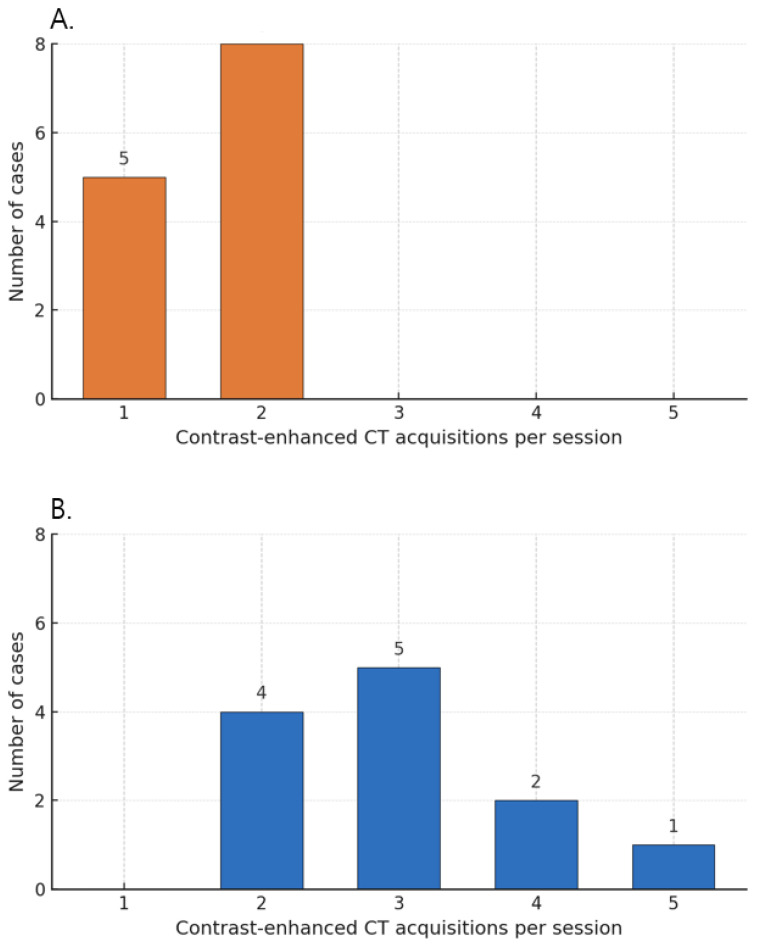
Number of contrast-enhanced CT acquisitions per session. (**A**) Conventional RFA group (n = 13). (**B**) Angio-CBCT group (n = 12). Note.—The following outlier cases were excluded from the analysis: (1) lipiodol-guided RFA after TACE, (2) cases in which only post-ablation confirmation CT was obtained, and (3) procedures performed with ultrasound guidance alone.

Across all procedures, total iodinated contrast material per session was capped at ≤250 mL. This cap directly constrained the number of contrast-enhanced CT acquisitions in the conventional CT group, where each contrast-enhanced acquisition required approximately 100–120 mL of IV contrast; Accordingly, all sessions in the conventional RFA group were restricted to ≤2 contrast-enhanced CT acquisitions per session due to the 250-mL contrast cap. In the angio CBCT group, despite the same 250-mL cap, selective intra-arterial injections via a 5-Fr catheter or microcatheter required approximately 20–40 mL per contrast-enhanced acquisition, allowing two to five acquisitions per session. These are summarized in Figure 7. Of the overall cohort (CBCT n = 14; conventional n = 20), the contrast-enhanced acquisitions analysis excluded prespecified outliers, yielding the analysis subset of n = 12 and n = 13, respectively.

### 3.6. Complications

One complication occurred. Following an otherwise uneventful RFA, the patient developed a post-procedural decline in hemoglobin and an increase in serum bilirubin. Inpatient abdominal CT showed no overt bleeding or biliary tract abnormality. Hepatic angiography was performed and demonstrated opacification of the bile duct, consistent with a hepatic artery–bile duct fistula with resultant hemobilia and hemoglobin loss. Coil embolization of the involved hepatic arterial branch was undertaken. The patient made a full recovery without sequelae; the patient’s Child–Pugh class was B. No procedure-related deaths occurred.

## 4. Discussion

In this single-center cohort, angiography suite cone-beam CT (angio-CBCT) guidance for RFA achieved 100% technical success and 0% early local recurrence in 14 patients, with one complication (CTCAE grade 3). These results suggest that embedding RFA within an angiography-CBCT workflow can mitigate several well-known limitations of conventional ultrasound- or CT-guided approaches.

We consider the principal advantages of angio-CBCT-guided RFA to be: (1) overcoming limitations inherent to ultrasound guidance; (2) overcoming limitations of conventional CT guidance, particularly with respect to contrast enhancement and contrast load; and (3) a marked increase in intraprocedural lesion conspicuity. We believe these procedural advantages contributed to the favorable outcomes observed—high technical success (100%) and low (indeed, zero) local recurrence (0%)—although our sample size was small.

Specifically, ultrasound guidance is constrained by poor conspicuity for small or isoechoic tumors and by limited acoustic windows for deep, subdiaphragmatic/dome lesions or targets obscured by lung, ribs, or bowel gas; steam/gas generated during ablation further creates a “blind period” that hampers real-time margin assessment [5,16,17,18]. These constraints translate into difficulty achieving the required ≥5-mm safety margin under conventional ultrasound (47.0% vs. 89.3% with ultrasound–ultrasound overlay fusion) and higher two-year local tumor progression (6.0% vs. 0.8%) [17]. Accordingly, ultrasound-only guidance may impair the technical success of RFA and the attainment of adequate safety margins. To mitigate these limitations, some form of CT guidance should be integrated into the procedural workflow to overcome the inherent shortcomings of ultrasound [16].

Ultrasound-based tumor targeting is technically straightforward but uncertainty-prone; to fully eliminate targeting ambiguity, “Contrast-enhanced cross-sectional imaging (CECT or angio-CBCT) at key steps is recommended when uncertainty must be minimized. In our workflow, we routinely obtain two acquisitions—(i) a pre-ablation scan immediately after needle placement to verify the needle–tumor relationship (to mitigate malposition-related complications and plan adequate margins), and (ii) an immediate post-ablation scan to assess the ablative margin. We acknowledge that some centers rely on post-ablation CT with re-ablation as needed to achieve margins; while reasonable, we favor pre-ablation verification because a baseline scan facilitates discrimination of peri-ablational hyperemia from true residual tumor.” Moreover, additional CECT is frequently necessary, including during electrode repositioning, when re-ablation is undertaken for an insufficient margin, or when initial targeting on ultrasound fails because the lesion is isoechoic or has indistinct tumor margin. Software-assisted margin confirmation—reported to outperform visual comparison—could be integrated into the described angio-CBCT workflow and may further enhance standardization; it was not evaluated here.

In conventional intravenous contrast–enhanced CT-guided approaches, each run typically requires approximately 100–120 mL of contrast, so practical constraints often limit enhancement to one or two acquisitions [6]. This was likewise the case in the conventional CT-guided RFA cohort at our institution (Figure 6). Consequently, intermediate steps may rely on noncontrast CT or ultrasound estimation, which does not fully eliminate uncertainty in targeting and margin assessment. In our workflow, each CBCT acquisition used 20–40 mL of iodinated contrast, permitting 2–3—and, when needed, more—enhanced intraprocedural acquisitions within a single session without exceeding typical contrast limits (Figure 6). This approach enables repeated assessment and comprehensive verification throughout the critical steps of curative-intent RFA.

Accordingly, angio-CBCT guidance enables repeatable, targeted three-dimensional acquisitions—including multiple low-volume enhanced runs—that are not constrained by acoustic windows and are less susceptible to steam-related obscuration. This facilitates reliable target visualization and on-table verification of electrode–tumor geometry and ablation margins, thereby minimizing procedural uncertainty and supporting confident endpoint determination.

Moreover, a key observation in our study is that intraprocedural lesion conspicuity increased markedly despite a lower contrast load. This was quantified as the ΔHU change between preprocedural imaging and intraprocedural acquisitions (CBCT group: median 290.1 HU [IQR, 197.5–414.1] vs. conventional group: −10.5 HU [IQR, −17.9–16.5]). (Figure 1) This increase in conspicuity translated into actionable, on-table decisions: electrode repositioning in 14.3% of cases and planned re-ablation in 21.4% (Table 2). Practically, the ability to “see better” at critical moments appears to tighten the imaging–therapy feedback loop, increasing the likelihood of achieving complete ablation within a single session and potentially reducing early local failure.

When compared with an institutional conventional RFA cohort (technical success 95%, local recurrence 10%), the angio-CBCT group showed numerically higher technical success and lower early local recurrence. These differences should be interpreted cautiously. (Our sample is small and was not powered for between-group testing; selection biases, operator learning effects, and heterogeneous follow-up likely influenced outcomes.) Still, the alignment between improved intraprocedural visibility (ΔHU), frequent real-time corrective actions (repositioning/re-ablation), and favorable early local control is internally consistent and hypothesis-generating.

Another advantage of the angio-CBCT workflow is opportunistic disease assessment during the procedure. In 7.1% of cases, we detected a new lesion that was not appreciated preprocedurally, allowing immediate modification of the treatment plan. Although such events were infrequent, the clinical value is high: detecting and addressing additional disease foci at the same sitting may prevent near-term retreatment and optimize resource use.

Safety outcomes were acceptable. We observed one grade 3 complication, which required subsequent management after the index RFA session rather than definitive treatment during the same sitting. While this single event precludes broad safety claims, the co-location of therapy and potential endovascular rescue (e.g., embolization for procedure-related vascular injury) remains an inherent benefit of performing RFA in the angiography suite; however, no such intraprocedural rescue was needed in our cohort. Notably, the comparator cohort (conventional RFA) had no recorded complications; however, cross-cohort inference is limited by small numbers and nonrandomized allocation.

There are several practical considerations to address. Although each CBCT run uses only 20–40 mL of iodinated contrast, cumulative exposure can become nontrivial when multiple acquisitions are performed for planning, needle checks, and post-ablation assessment; per-patient contrast budgets and renal risk should therefore be monitored, with prespecified run number/timing to balance image quality and total dose. Implementing this workflow also requires anesthesia resources and suite time, and would benefit from standardized protocols (e.g., acquisition timing, reconstruction parameters, acceptance criteria for target conspicuity, and objective triggers for re-ablation) to enhance reproducibility and scalability.

This study has limitations. It is a small, single-center, nonrandomized comparison between contemporaneous cohorts, with limited and heterogeneous follow-up durations. Quantitative ΔHU analysis was feasible only in subsets of each cohort (n = 12 and n = 13), reducing statistical power. Baseline differences between cohorts were not adjusted, introducing potential residual confounding. These factors limit the strength and generalizability of our comparative statements. Future studies—recognizing practical constraints—should be adequately powered, prespecify and standardize imaging/contrast protocols, and include analytic adjustment for baseline imbalances between cohorts. “Lack of software-based margin evaluation may affect reproducibility and generalizability.”

## 5. Conclusions

In conclusion, angio-CBCT-guided RFA for HCC was feasible and demonstrated excellent technical performance and early local control in our cohort. The quantified increase in intraprocedural lesion conspicuity appears to facilitate on-table corrective actions that may underlie these outcomes. Prospective studies with larger samples, standardized imaging/ablation endpoints, complete dose and contrast accounting, and longer oncologic follow-up are warranted to define where angio-CBCT adds the greatest value relative to conventional guidance.

## Figures and Tables

**Table 1:** Patient demographics and concise tumor summary.

Characteristic	Angio CBCT	Benchmark
Total patients, n	14	20
Sex—Male, n (%)	8 (57.1%)	17 (85.0%)
Sex—Female, n (%)	6 (42.9%)	3 (15.0%)
Median age, years—median [range]	71 [52–85]	70 [52–82]
Child–Pugh class—A, n (%)	12 (85.7%)	20 (100.0%)
Child–Pugh class—B, n (%)	2 (14.3%)	0 (0.0%)
Child–Pugh class—C, n (%)	0 (0.0%)	0 (0.0%)
Tumor type—HCC, n (%)	14 (100.0%)	20 (100.0%)
Largest tumor, cm—median [range]	1.6 [0.9–2.5]	1.9 [0.5–3.2]
Lesions—1/≥2, n (%)	12 (85.7%)/2 (14.3%)	18 (90.0%)/2 (10.0%)
Tumor ≥3 cm, n (%)	0 (0.0%)	1 (5.0%)

Note—Data are n (%) unless otherwise indicated; continuous variables are median [range]. Abbreviations—CBCT = cone-beam CT; HCC = hepatocellular carcinoma.

**Table 2:** Procedural Outcomes in the Angio CBCT-Guided Cohort.

Outcome	n/N	%
Technical success	14/14	100.0
Local recurrence	0/14	0.0
Complications (overall)	1/14	7.1
CTCAE grade (v6.0)	Grade 3	
Intra-procedural refinements during the session		
Target repositioning	2/14	14.3
Immediate re-ablation	3/14	21.4
New lesion detected intra-procedurally	1/14	7.1

Note.—Data are n/N (%). Percentages are rounded to one decimal place. Abbreviations: CBCT = cone-beam computed tomography.

**Table 3:** Outcomes by treatment group (Angio CBCT RFA vs. Conventional RFA).

Outcome	Angio CBCT RFA n/N (%)	Conventional RFA n/N (%)	*p* Value
Imaging follow-up, median [IQR], days	460 [205–636]	967 [801–1076]	-
Technical success	14/14 (100.0%)	19/20 (95.0%)	1.000
Local recurrence	0/14 (0.0%)	2/20 (10.0%)	0.501
Complication	1/14 (7.1%)	0/20 (0.0%)	0.412
Local control (KM), 12 mo	100% (95% CI NE)	100% (95% CI NE)	-
Local control (KM), 24 mo	100% (95% CI NE)	94.1% (95% CI 65.0–99.1)	-

Footnote: 95% CI NE = not estimable due to zero events; RFA = radiofrequency ablation. Patients were censored at last imaging or 31 October 2025 (administrative censoring); deaths were not modeled as competing events.

## Data Availability

The data presented in this study are available on reasonable request from the corresponding author. The data are not publicly available due to privacy and institutional ethical restrictions.
